# Chromosomal position effect influences the heterologous expression of genes and biosynthetic gene clusters in *Streptomyces albus* J1074

**DOI:** 10.1186/s12934-016-0619-z

**Published:** 2017-01-04

**Authors:** Bohdan Bilyk, Liliya Horbal, Andriy Luzhetskyy

**Affiliations:** 1 PharmBioTec GmbH, Science Park 1, 66123 Saarbrücken, Germany; 2 Helmholtz-Institute for Pharmaceutical Research Saarland, Campus, Building C2.3, 66123 Saarbrücken, Germany; 3 Department of Pharmaceutical Biotechnology, Saarland University, 66123 Saarbrücken, Germany

**Keywords:** *Streptomyces albus* J1074, Heterologous expression, Chromosomal position effect, φC31-attachment site

## Abstract

**Background:**

Efforts to construct the *Streptomyces* host strain with enhanced yields of heterologous product have focussed mostly on engineering of primary metabolism and/or the deletion of endogenous biosynthetic gene clusters. However, other factors, such as chromosome compactization, have been shown to have a significant influence on gene expression levels in bacteria and fungi. The expression of genes and biosynthetic gene clusters may vary significantly depending on their location within the chromosome. Little is known about the position effect in actinomycetes, which are important producers of various industrially relevant bioactive molecules.

**Results:**

To demonstrate an impact of the chromosomal position effect on the heterologous expression of genes and gene clusters in *Streptomyces albus* J1074, a transposon mutant library with randomly distributed transposon that includes a β-glucuronidase reporter gene was generated. Reporter gene expression levels have been shown to depend on the position on the chromosome. Using a combination of the transposon system and a *φC31*-based vector, the aranciamycin biosynthetic cluster was introduced randomly into the *S. albus* genome. The production levels of aranciamycin varied up to eightfold depending on the location of the gene cluster within the chromosome of *S. albus* J1074. One of the isolated mutant strains with an artificially introduced attachment site produced approximately 50% more aranciamycin than strains with endogenous *attB*s.

**Conclusions:**

In this study, we demonstrate that expression of the reporter gene and aranciamycin biosynthetic cluster in *Streptomyces albus* J1074 varies up to eightfold depending on its position on the chromosome. The integration of the heterologous cluster into different locations on the chromosome may significantly influence the titre of the produced substance. This knowledge can be used for the more efficient engineering of Actinobacteria via the relocation of the biosynthetic gene clusters and insertion of additional copies of heterologous constructs in a suitable chromosomal position.

**Electronic supplementary material:**

The online version of this article (doi:10.1186/s12934-016-0619-z) contains supplementary material, which is available to authorized users.

## Background

During the past few decades, streptomycetes have been extensively employed for the heterologous expression of bioactive natural products. Heterologous expression contributes substantially to the discovery and characterisation of secondary metabolites. The increase in the number of sequenced microbial genomes demonstrates that most of the biosynthetic clusters remain silent under standard laboratory conditions. The expression of silent gene clusters in a well-characterised heterologous host is a validated approach for the discovery of new natural products [1]. However, insufficient yields of heterologously produced molecules often prevent their further purification and characterisation. Currently, several *Streptomyces* strains have been utilised as hosts for the production of natural products. The most widely used are *Streptomyces coelicolor, Streptomyces avermitillis* and *Streptomyces albus* J1074 [2]. However, the production level of the heterologously produced compounds often needs to be further optimised. Industrially relevant titres of some natural products may reach the multi-gram per litre scale (e.g., ≥10 g/L erythromycin; ≥30 g/L tetracycline; ≥80 g/L salinomycin; personal communication with Prof. Hrvoje Petkovic, Ljubljana University), whereas the yields of natural products in heterologous hosts remain in the milligram range [2]. One of the factors significantly influencing the expression of genes and biosynthetic gene clusters is their location on the chromosome, the so-called ‘chromosomal position effect’. This term describes differences in the expression of a gene depending on its chromosomal location. Studies of gene expression in *Saccharomyces cerevisiae* demonstrated that the expression of heterologous genes was repressed when integrated at silent loci, indicating that repression occurred due to a positional effect [3]. In another study [4], it was shown that an *Aspergillus nidulans* mutant, defective in the system responsible for histone methylation, activates silent secondary metabolite clusters.

The position effect can influence not only the expression level of a native gene after spontaneous translocations, but also transgene expression after insertion into different regions of a genome, leading to changes in heterologous production. It was demonstrated that variations in β-galactosidase activity can reach 300-fold in response to translocations of the β-galactosidase gene in the *Escherichia coli* chromosome [5]. However, Schmid and Roth demonstrated [6] only a threefold variation in the expression level of the *his* operon cluster randomly distributed within the *Salmonella typhimurium* chromosome. Additionally, *Lactobacillus lactis* mutants showed a threefold difference in the levels of *gus(a)* expression [7].

The main factors causing such variability in gene expression are: (i) the level of DNA compactization [8]; (ii) variations in the promoter strength; and (iii) the distance to the origin of replication [7]. The first two factors are more critical for eukaryotic organisms, which have high levels of DNA compactization and greater variation in promoter strengths, which may influence a downstream heterologous gene. By contrast, the distance to the replication origin is the major factor of variability in gene expression in prokaryotic cells, because they contain a single origin of replication per genophore. Thus, a gene placed closer to the origin of replication is replicated before a gene located near the terminus and therefore has an operative increase in gene dosage [9].

Despite the importance of streptomycetes as producers of natural products, the position effect has not yet been investigated in them. The representatives of this genus have a complex life cycle. These bacteria colonise the environment by growing branching, multigenomic hyphae while simultaneously forming unigenomic spores to achieve dispersion. Recent studies identified a novel protein specific to Actinobacteria that is responsible for DNA compactization, chromosome segregation and antibiotic production [10]. In other studies [11], a histone-like protein was characterised that can structurally couple changes in DNA conformation and transcription in response to stress. Such complexity in nucleoid organisation leads to variations in expression of different DNA regions and causes a chromosomal position effect. However, to date, a lack of efficient tools for the exploration of streptomycetes genomics has limited any attempts to evaluate the impact of this factor on the expression of heterologous genes and clusters.

Here, we extend previous studies on the chromosomal position effect to the mycelial microorganism *Streptomyces albus* J1074, which is a typical representative of the *Streptomyces* genus and is well known for its outstanding potential as a host for the heterologous production of bioactive small molecules [12]. During the past decade, *S. albus* J1074 has been used for the heterologous expression of various antibiotic biosynthetic clusters, e.g. thiocoraline, cyclooctatin and steffimycin. Numerous genetic tools have recently been developed for the strain, making its genome engineering rather straightforward [13–15].

To evaluate the impact of the chromosomal position effect on heterologous expression in the genome of *S. albus* J1074, a transposon mutant library with randomly distributed *gus(a)*, the β-glucuronidase reporter gene, was generated. The expression of the reporter gene in recombinant strains was analysed, and various factors influencing the expression of the *gusA* gene are discussed. In addition, using a combination of a transposon system and the *φC31*-based vector, the aranciamycin biosynthetic cluster was introduced randomly into the *S. albus* genome. Analysis of aranciamycin producers also demonstrated an eightfold variation in the production level of the antibiotic depending on its chromosomal location. One of the isolated mutant strains with an artificially introduced attachment site produced 53% more aranciamycin than strains with endogenous *attB*s.

## Results and discussion

### Impact of the chromosomal position effect on gene expression

**Fig. 1:**
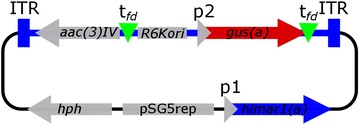
The map of the plasmid containing *gus(a)* in the transposon, pALG. Plasmids contains following features: *himar1(a)*—synthetic transposase gene, under control of p1—*tipA*p, thiostrepton inducible promoter; *gus(a)*—reporter gene of glucuronidase; p2—*ermE*p1, promoter 1 of erythromycin resistance gene; *tfd* terminator of *fd*-phage, *pSG5rep* actinomycetes temperature-sensitive replicon; *aac(3)IV* apramycin resistance marker, *hph* hygromycin resistance marker, ITR inverted terminal repeats; *R6Kori* origin for rescue cloning

**Fig. 2:**
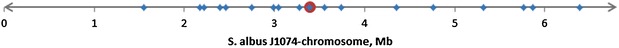
Distribution of insertion loci for pALG-transposons in *S. albus* J1074. *Blue rhombs* represent positions of the mapped transposon insertions; *red circle* represents position of *oriC* of *S. albus*-chromosome

To examine an impact of the chromosomal position effect on the expression of heterologous genes in *S. albus* J1074, a transposon harbouring the reporter gene was constructed (Fig. 1). The transposon contained the *gus(a)* reporter gene, encoding β-glucuronidase under the control of the *ermE*p1 promoter [16]. The activity of β-glucuronidase can be spectrophotometrically measured. The *gus(a)* gene is flanked by two *fd*-phage terminators [17], which prevent transcription in both directions with an efficiency of up to 85% (Additional file 1: Figure S1). The apramycin resistance gene was cloned upstream and in the opposite direction to *gus(a)* to exclude any impact on expression of the reporter gene. Then the transposon was cloned into the plasmid containing synthetic gene of Himar1-transposase and temperature sensitive pSG5 replicon. The vector was introduced into genome *S. albus* J1074 and library of transposon mutants was generated as described previously [14]. Eighteen mutants from this library with the randomly distributed reporter gene were isolated and analysed. The insertions were mapped on the chromosome using rescue cloning, as described elsewhere [14] (Table 1; Fig. 2). All identified insertions were localised in a core region of the chromosome. A measurement of the GusA activities was made after 48 h of growth (Table 1; Fig. 4). The average value of activity was 8.6 U/mg, and eight of the analysed mutants (44%) had activities that lay within this range (mutants # 1, 2, 4, 5, 14, 16, 17 and 18). Five mutants (28%) had activity values above average, and another five mutants (28%), located mostly in the centre of the chromosome, and had values below average. The variation in the reporter gene expression was six-fold (the lowest value observed was 2.7 U/mg, and the highest was 17.3 U/mg), which was comparable to results described for other prokaryotes, where two- to threefold variations in activity have been observed [6, 7, 18].

**Table 1:** Loci of pALG-transposon insertions in *S. albus* J1074 identified by rescue plasmid sequencing

M	Locus (XNR)	Gene function	GUS activity (U/mg)	St. dev. (U/mg)	Expression level (RPKM)
1	1302	Urease subunit α 1	8.2	0.6	1.9
2	1853	Bi-functional transferase/deacetylase	7.7	0.4	2.2
3	1880	Phosphatase	15.4	2.1	42.5
4	2035	Cons. hyp. prot./biosynthesis docking scaffold prot.	9.1	1.3	5.7
5	2089	Integral membrane protein	8.1	0.1	24.7
6	2358	Reg. prot./MerR-family transcriptional reg.	11.8	1.1	22.7
7	2599	Predicted protein/adenylate cyclase	17.3	1.8	3.5
8	2683	Beta-lactamase	3.4	1.7	31.7
9	2883	TetR-family transcriptional regulator	6.2	1.2	6.0
10	2981	Integral membrane protein	3.5	0.5	102.6
11	3133	Peptidase C14 caspase catalytic subunit	3.3	0.1	97.1
12	3300	Conserved hypothetical protein	2.7	0.9	2.3
13	3826	Conserved hypothetical protein/DNA-binding prot.	9.9	1.1	27.5
14	4204/5	IGR (intergenic region) btw. two predicted proteins	8.6	1.1	25.4
15	4675/6	IGR btw. cons. hyp. protein and phospholipase	14.7	1.5	19.6
16	5073/4	IGR btw. transmembr. transporter and integral membr. prot.	8.8	1.3	3.5
17	5168	Glycerol kinase 1/2	7.8	1.0	27.9
18	5663	Succinate dehydrogenase flavoprotein subunit	8.0	1.3	29.9

**Fig. 3:**
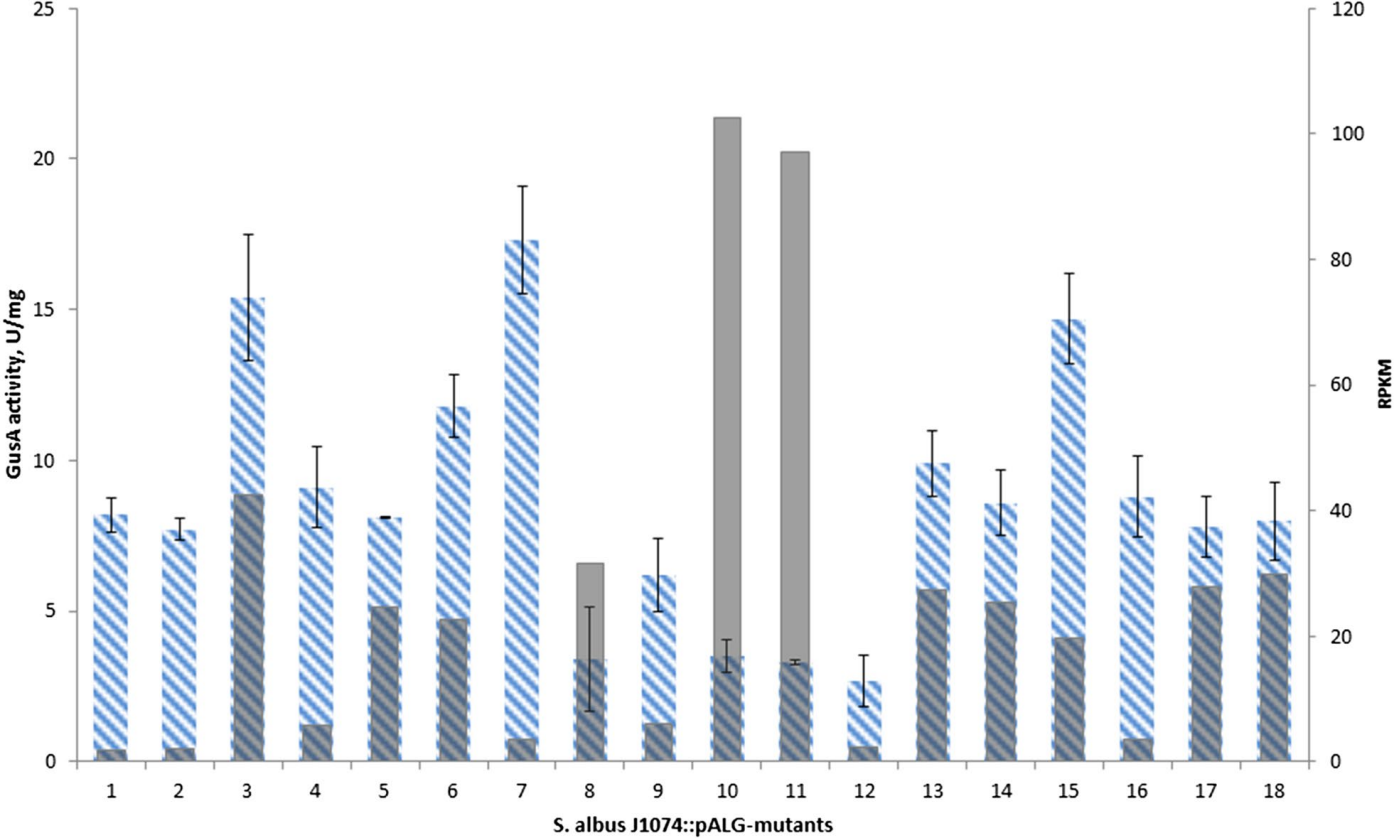
The comparison of GusA-activity levels with expression level of adjacent genes (correlation coefficient is equal—0.34). *Blue columns* correspond to values of GusA-activity. Strains were grown for 48 h at 28 °C; *light grey columns* correspond to RPKM after 48 h of cultivation at 28 °C. Mutants are placed according to location of their transposons on the chromosome

Owing to the isolation of *gus(a)* by the two terminators, the activity of the local promoters should not have had a significant impact on its expression. To confirm this, the GusA activities of the mutants were compared with *S. albus* J1074 RNA-seq data after 48 h of cultivation in liquid medium. The RPKM (reads per kilobase per million reads) values of the genes, which are located in the genome of *S. albus* J1074 upstream and in the same orientation as *gus(a)*, were analysed. The results demonstrated that, in the obtained transposon mutants, local promoters had a minor effect on the level of *gus(a)* expression, e.g., mutant 10, which exhibited one of the lowest levels of GusA activity, was flanked upstream by the region with a high RPKM value (Fig. 3). Concurrently, mutants with a relatively low RPKM of flanking genes (e.g. mutants 7 and 15) were characterised by a high GusA activity level (Fig. 3).

### Chromosomal position effect does not correlate with distance to oriC

Previous studies on the position effect in *E. coli* and *Salmonella* demonstrated that variations in the expression level of reporter genes are caused mainly by gene dosage [6, 19–21]. Unlike most bacteria that possess a circular chromosome, streptomycetes carry a linear chromosome with a centrally located *oriC*. Thus, if the gene dosage plays a significant role in the expression level of heterologous genes, the GusA activity in mutants with insertions localised closer to the chromosome centre should be higher than in those with insertions localised closer to the chromosome ends. However, plotting the GusA activities with positions of the reporter gene showed that mutants with integration near the *oriC* demonstrated GusA activity below average (mutants 8, 9, 10, 11 and 12), whereas strains with an insertion located some distance from the *oriC*, closer to centres of the chromosomal shoulders, demonstrated above-average results (mutants 3, 6 and 15).

### Relocation of the site for gene cluster integration

**Fig. 4:**
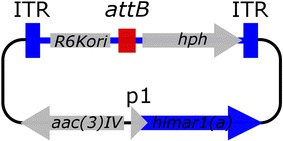
The map of the plasmid containing phage attachment site in the transposon, pAHT. Plasmids contains following features: *himar1(a)*—synthetic transposase gene, under control of p1—*tipA*p, thiostrepton inducible promoter, *aac(3)IV* apramycin resistance marker, *hph* hygromycin resistance marker, ITR inverted terminal repeats, *R6Kori* origin for rescue cloning, *attB* attachment site of *φC31*-phage

The obtained results demonstrated that the translocation of the heterologous construct may significantly influence its expression. This result should also be valid for more complex genetic structures, such as an antibiotic biosynthetic gene cluster. It is difficult to deliver antibiotic biosynthetic gene clusters into a streptomycetes chromosome via a transposon, due to their large size. Most of the vectors carrying biosynthetic gene clusters are integrated into the chromosome via the phage *φC31* integration system. Thus, we translocated the *attB* attachment site of *φC31* into various locations of the *S. albus* J1074 chromosome. A transposon with an attachment site of the *φC31*-phage was constructed and cloned into a non-replicative vector containing *himar1(a)*, the synthetic transposase gene. The obtained plasmid pAHT (Fig. 4) did not require any additional induction steps or curing of the plasmid backbone, because the exconjugants obtained already contained a copy of the transposon inserted in the genome. This vector was than introduced into *S. albus* SAM3 (Δ*attB·*Δ*pseB*4), lacking both endogenous *attB* sites [22], by intergeneric conjugation from *E. coli*.

**Fig. 5:**
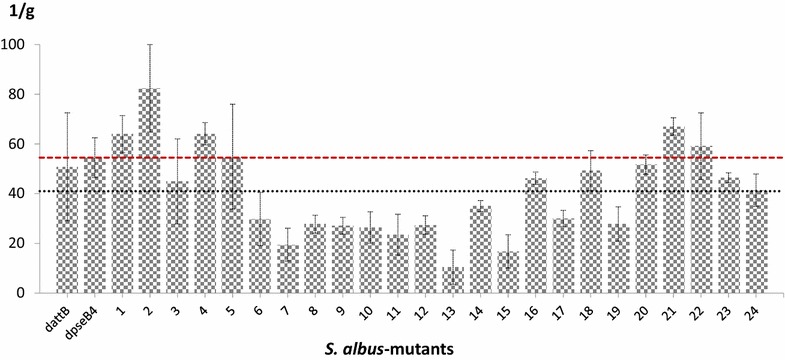
Relative concentration of aranciamycin in culture media of heterologous aranciamycin producers. dattB—*S. albus* SAM1(ΔattB)::p412C06; dpseB4—*S. albus* SAM2(ΔpseB4)::-p412C06; 1-24—*S. albus* SAM3 (ΔattB·ΔpseB4)::pAHT::p412C06-mutants*. Dotted black* *line* corresponds to average production of aranciamycin by transposon mutant strains; *dotted red line* corresponds to average production of aranciamycin by control strains with one integration site. Strains were grown for 120 h at 28 °C. Relative concentration of aranciamycin was calculated as peak area per gram of dry mass

The aranciamycin gene cluster was integrated into artificially distributed *attB*s. This 35.9-kb large cluster was isolated from *Streptomyces echinatus* [23] and was previously expressed in different streptomycetes [24]. The cosmid with the aranciamycin cluster was introduced into twenty-four transposon mutants. In this way, a library of strains carrying the aranciamycin biosynthetic cluster at different chromosomal positions was obtained. As a control, two strains containing naturally located *attB*s (Δ*attB* and Δ*pseB*4 [22]) were used. Altogether, the mutants demonstrated an eightfold variation in aranciamycin production. Such a variation in the expression level of a heterologous cluster correlated with results obtained for *gus(a)*-gene expression described herein. The average production of aranciamycin for the mutant strains was 40.53 U/mg. However, in this case, we observed stronger variation in the results: only one of the 24 mutants produced in the range of 40.53 ± 10% (mutant 24). Thirteen mutants (54%) demonstrated below-average results, and 10 mutants (42%) evinced above-average results (Fig. 5). The production of aranciamycin from four artificially integrated *attB*s was more than 20% above the average production from endogenous attachment sites (Fig. 5, mutants 1, 2, 4 and 21). The yield of aranciamycin in one of those mutants (mutant 2) was 56% higher than in the control strains. This result demonstrated that the translocation of the cluster may result in significant variation in antibiotic production.

These data suggest that the central regions of the chromosomal “shoulders” are more preferable loci for the expression of heterologous constructs, because GusA activity in mutants with these integrations was higher than average. Given that streptomycetes are well-established cell factories for the biosynthesis of natural products, it is important to study the influence of chromosomal localisation on the expression of biosynthetic gene clusters.

Indeed, the eightfold variation in antibiotic production was observed in the library of mutants carrying corresponding gene clusters in different locations of the chromosome. Four of the 24 isolated mutants demonstrated more than a 20% increase in antibiotic production, and one of these mutants showed a 56.5% increase in the yield of aranciamycin (82.4 U/mg) compared with the average production of the control strains (52.65 U/mg).

Thus, the proposed approach can be used in combination with other methods that are employed for the optimisation of the heterologous production of natural products. This approach can be used not only for translocation of the attachment site, but also to introduce additional integration sites into the genome, thus resulting in an increase in the number of clusters per genome [25]. We believe that the genetic tools described herein will find wide applications in the construction and manipulation of actinobacterial heterologous hosts.

## Conclusions

Our study demonstrates the effect of the chromosomal position effect on a heterologously expressed reporter gene and on more complex genetic constructs, such as antibiotic biosynthetic gene clusters in *S. albus* J1074. Based on this phenomenon, we developed an approach that can be used for the construction of host strains with improved heterologous production features.

## Methods

### Bacterial strains and cultivation conditions

The cultivation of the *S. albus* strains was conducted on a soy-mannitol agar or in a liquid TSB medium at 29 °C. The cultivation of *E. coli* was conducted in an LB medium at 37 °C. The intergeneric conjugation of the plasmids was performed as previously described [26]. The library of transposon mutants was obtained as described elsewhere [14].

### Recombinant DNA techniques

Lambda-red-mediated recombination was conducted as described in the standard protocol [27]. The isolation of genomic DNA from *S. albus* strains was performed according to a previously described method [26]. PCR using Phusion polymerase (Thermo Scientific), separation of DNA by agarose gel electrophoresis and Southern blot hybridisation were performed as previously described [28]. DNA fragments and PCR products were purified using PROMEGA™ kits. Oligonucleotides were synthesised by Eurofins MWG Operon. DNA sequencing was performed by GATC-Biotec.

### Isolation of rescue plasmids

Chromosomal DNA was isolated from the desired strains and digested with *Sac*II for pALG transconjugants, respectively. The fragments were then self-ligated and transformed into *E. coli* Transformax cells. The transformants were selected based on their resistance to apramycin. The obtained plasmids were isolated and sequenced using the P1-pALG-ch primer.

### Plasmid construction

The plasmids and primers used in this study are listed below (see Additional file 1). The DNA manipulations and cloning procedures were performed according to standard protocols [29], and the plasmid constructs were confirmed by DNA sequencing.

#### Construction of pALG

The *gus(a)* gene was amplified using the pSET*gus(a)* plasmid as a template, the forward primer, Fr-*X*I-*e*p1-*gus(a)*, carrying the *Xba*I site and *ermE*p1, a *Saccharopolyspora erythraea ermE*p1 gene promoter and the reverse primer, Rs-*M*I-t*fd*-*gus(a)*, carrying the *fd*-phage terminator and the *Mun*I site. The PCR fragment was ligated into p*Nhe*I*aac* [14] linearised by *Xba*I and *Mun*I leading to p11-8. The *aac(3)IV* gene was amplified using pIJ773 as a template, the forward primer, Fr-*E*RI-t*fd*-*aac*, carrying the *fd* terminator and the reverse primer, Rs-*E*RI-*aac*. Both primers carry the *Eco*RI site. The fragment was cloned into the *Eco*RI site of p11-8 replacing an existing *aac(3)IV*, resulting in p11-8*aac*t*fd*. The *Pvu*II fragment of p11-8*aac*t*fd*, containing the transposon, was ligated to pALHim linearised with *Eco*RV, to yield pALG (Fig. 1).

#### Construction of pAHT

The *hph* gene was amplified using pAL1 as a template, with Fr-*M*I-*attB*-*hph* as the forward primer, carrying *attB* and the *Mun*I restriction site, and Rs-*X*I-*hph* as the reverse primer carrying the *Xba*I site. The amplified fragment was cloned into the *Mun*I and *Xba*I sites of pTn5Oks resulting in pTn5Oks*attBhph*(II). To construct a backbone of the transposon vector, the hygromycin resistance gene in p31Him was replaced by the apramycin resistance gene using λ-red-mediated recombination [30]. The primers Fr-*hph*/*aac(3)IV* and Rs-*hph*/*aac(3)IV* were used to amplify the fragment for recombination. The obtained plasmid, pAHS, was linearized by *Eco*RV and ligated with the transposon from the pTn5Oks*attBhph*(II) plasmid, giving pAHT (Fig. 4).

### Measurement of glucuronidase activity

After a stationary phase of growth was reached, 10 mL of the main culture was transferred into 15 mL-conical centrifuge tubes. Mycelium was pelleted by centrifugation, the supernatant was discarded and the samples were dried for 24 h at 65 °C. After 24 h, the conical centrifuge tubes with dry mycelium were weighed. The spectrophotometric measurement of glucuronidase activity was carried out as previously described [29].

### Strain cultivation and HPLC analysis

The *S. albus* strains containing the aranciamycin biosynthetic cluster were grown in 20 mL of a TSB medium for 48 h at 30 °C. Then, 200 μL of preculture were transferred into 15 mL of a NL5 + 1% yeast extract medium and cultivated for an additional 120 h at 30 °C. After cultivation, 5 mL of the mycelia were harvested by centrifugation for 30 min at 2000 rcf. The supernatant was transferred into a new Falcon tube and mixed with 5 mL of ethyl acetate and rotated on a rotator for 30 min. Then, the samples were centrifuged at 4000 rpm for 10 min, and the upper phase of the samples was transferred to glass vials and dried under N_2_. The dry pellet was dissolved in acetonitrile and centrifuged at 20,000 rcf for 10 min to remove the cell debris. Then, 80 μL of the extracts were transferred into HPLC vials and analysed by HPLC.

The HPLC–ESI–MS-UV–Vis analysis was performed on a Dionex Ultimate 3000 HPLC system (Thermo Fisher Scientific) that was connected to an ESI–MS Amazon mass spectrometer (Bruker). The HPLC system was equipped with a 100 × 2.1 mm, 1.7-µm BEH C18 column (Waters); a 50 × 2.1 mm, 1.7-µm BEH C18 column (Waters); or a 100 × 2 mm, 2.5-µm Luna C18 column (Phenomenex), depending on the method. For unknown extracts, an 18-min gradient was performed, mainly on the long column, whereas a 9-min gradient was used for the prepurified or known extracts. All gradient methods began with 5% B and increased to 95% B over 9 or 18 min. Solvent A consisted of H_2_O + 0.1% formic acid, whereas solvent B contained ACN+ 0.1% formic acid. The flow rate for the BEH C18 columns was 0.6 mL/min, whereas the flow rate for the Luna C18 column was 0.4 mL/min; UV–Vis detection was performed from 210 to 600 nm. Most of the time, the ESI–MS was used in alternating mode (positive and negative). Only a full MS scan or an additional scan with MS^2^ data was recorded, depending on the method. Both HPLC and MS systems were combined using the Hystar program (Bruker). As such, the standard LC–MS experiments could be selected as supermethods. These experiments included the selected column (Luna = Luna C18; 100 = BEH C18 100 mm; 50 = BEH C18 50 mm), gradient (9, 18 min) and MS mode (MS only, ms2 posneg).

High-resolution ESI–MS was measured using a Maxis Q-Tof 4G (Bruker) or an Orbitrap LTQ (Thermo Fisher Scientific) mass spectrometer connected to the same HPLC system used for standard LC–MS.

## Additional file

**Additional file 1: Figure S1.** Glucuronidase activity in cell lysates of recombinant *S. albus* strains. **Table S1.** Plasmids used in this work. **Table S2.** Primers used in this work.

## Abbreviations

RPKM: reads per kilobase of transcript per million reads
Cons. hyp. prot.: conservative hypothetical protein
Reg. prot.: regulatory protein
IGR: intergenic region
Membr. prot.: membrane protein
ME: mosaic end
ITR: inverted terminal repeat
