# Supplementary material for: Chromosomal position effect influences the heterologous expression of genes and biosynthetic gene clusters in Streptomyces albus J1074
Source: Microb Cell Fact. 2017 Jan 4;16:5. doi: 10.1186/s12934-016-0619-z (PMC5209838; doi:10.1186/s12934-016-0619-z)
Supplement: Supplementary file 1 — Additional file 1: Figure S1. Glucuronidase activity in cell lysates of recombinant S. albus strains. Table S1. Plasmids used in this work. Table S2. Primers used in this work. [file 12934_2016_619_MOESM1_ESM.docx]

**Chromosomal position effect influences the heterologous expression of genes and biosynthetic gene clusters in *Streptomyces albus* J1074**

Bohdan Bilyk^1,2^, Liliya Horbal^3^, Andriy Luzhetskyy^1,2,3^*

1. PharmBioTec GmbH, Science Park 1, 66123 Saarbrücken, Germany.

2. Helmholtz-Institute for Pharmaceutical Research Saarland, Campus, Building C2.3, 66123 Saarbrücken, Germany.

3. Department of Pharmaceutical Biotechnology, Saarland University, 66123 Saarbrücken, Germany.

Autors‘ e-mails: [B.Bilyk@leeds.ac.uk](mailto:B.Bilyk@leeds.ac.uk), [l.horbal@mx.uni-saarland.de](mailto:l.horbal@mx.uni-saarland.de), [a.luzhetskyy@mx.uni-saarland.de](mailto:a.luzhetskyy@mx.uni-saarland.de)

| **Table SM1**  **Plasmids used in this work** | | |
| --- | --- | --- |
| **Name** | **Description** | **Reference or Source** |
| pSET152*gusA* | pSET152-derivative, containing *gus(a)* | 26 |
| pIJ773 | pBluescript II SK-derivative, containing *aac(3)IV* and *ori*T flanked by two *loxP-*sites | 27 |
| pNheIaac | Synthetic plasmid containing R6Kγ origin and two *rox* sites framed by two ITRs (inverted terminal repeats) | 14 |
| pTn5Oks | PCR-derivative containing R6Kγ origin flanked by two MEs (mosaic ends) and two ITRs | Shine Gene, PRC |
| pALHim | Replicative vector for actinomycetes containing pSG5-rep, *ori*T and *himar1(a)* gene under *tipA* promoter | 14 |
| pNLTn5 | Replicative vector for actinomycetes containing pSG5-rep, *ori*T, and *tn5* gene under *tipA* promoter | 13 |
| p31Him | Suicide vector for actinomycetes containing *himar1(a)* gene under *phi*C31-integrase promoter | 14 |
| pAHS | Plasmid derived from p31Him with *hph* replaced by *aac(3)IV* | This work |
| pALG | pALHim-based plasmid containing minitransposon with *aac(3)IV*, *gusA* framed by two *fd* terminators, R6Kγ origin, flanked by ITRs | This work |
| pAHT | Plasmid derived from pAHS containing minitransposon with *hph*, *phi*C31-*attB*, R6Kγ origin, flanked by ITRs | This work |

| **Table SM2**  **Primers used in this work** | | |
| --- | --- | --- |
| **Name** | **Primer sequence (in 5’->3’)** | **Features** |
| Fr-*X*I-*e*p1-*gusA* | ccccc*tctaga*GTTGTGGGCTGGACAATCGTGCCGGTTGGTAG GATCCAGCGatgctgcggcccgtcga aaccc | *Xba*I (in italics); *ermE*p1 (in capitals) |
| Rs-*M*I-t*fd*-*gusA* | ggggg*caattg*AAAAAAAAAGGCTCCAAAAGGAGCCTTTAAtcactgcttcccgccctgctg | *Mun*I (in italics); *fd* terminator (in capitals) |
| Fr-*E*RI-t*fd*-*aac* | ccccc*gaattc*AAAAAAAAAGGCTCCAAAAGGAGCCTTTAAcacggtaactgatgccgtatt | *Eco*RI (in italics); *fd* terminator (in capitals) |
| Rs-*E*RI-*aac* | ccccc*gaattc*ggaataggaacttatgagctc | *Eco*RI (in italics); |
| Fr-*M*I-*attB*-*hph* | ccccc*caattg*CGGGTGCCAGGGCGTGCCCTTGGGCTCCCCGGGCGCGTACccgtatttgcagtaccagcgt-3 | *Mun*I (in italics), *attB* (in capitals) |
| Rs-*X*I-*hph* | ccccc*tctaga*gaataggaacttcggaatagg | *Xba*I (in italics) |
| Fr-*hph*/*aac(3)IV* | gaataggaacttcggaataggaacttcaggcgccgggg*gggaataggaacttatgagct* | pIJ773-homology regions (in italics) |
| Rs-*hph/aac(3)IV* | ccgtatttgcagtaccagcgtacggcccacagaatgatg*ggttcatgtgcagctccat* |  |

**Figure 1S. Glucuronidase activity in cell lysates of recombinant *S. albus* strains. Activity in *S. albus* containing *gusA* under the control of the p21 synthetic promoter (left) and under p21 insulated from the *gusA* gene by the fd-terminator (right).** The *gusA* gene was fused with the p21 promoter and with the p21 followed by *fd* terminator respectively using PCR. Obtained 1.9 kb fragments were then cloned into the pGUS plasmid using *Spe*I and *Eco*RV restriction sites. As a result two constructs were obtained. In one construct the *gusA* was expressed directly from p21 and in the second vector the expression was blocked by *fd* terminator. These two plasmid were introduced into the genome of *S. albus* J1074 by conjugation and exconjugants were obtained. Then, after 2 days growth in TSB media the GUS activities of obtained transformants were measured.
